# Severity and Clinical Outcomes of Pediatric Burns—A Comprehensive Analysis of Influencing Factors

**DOI:** 10.3390/jpm14080788

**Published:** 2024-07-25

**Authors:** Alexandra Toma, Dragoș Voicu, Constantin Popazu, Daniela Mihalache, Oana Duca, Dumitru Marius Dănilă, Dan Mircea Enescu

**Affiliations:** 1 Faculty of Medicine and Pharmacy, “Dunărea de Jos” University of Galați, 800201 Galați, Romania; 2 Emergency Clinical Hospital of Brăila, 810325 Brăila, Romania; 3 “Saint John” Emergency Clinical Hospital for Children, 800487 Galați, Romania; 4 “Carol Davila” University of Medicine and Pharmacy, 020021 București, Romania; 5 “Grigore Alexandrescu” Emergency Clinical Hospital for Children, 011743 București, Romania

**Keywords:** pediatric burns, burn severity, demographic factors, environmental factors, burn causative agents, clinical outcomes, prevention strategies, treatment protocols.

## Abstract

(1) Background: Burn injuries in children present a significant public health concern due to their potential for severe physical and psychological impact. (2) Methods: This study investigates the determinants of pediatric burn severity by analyzing the interplay of demographic and environmental factors. Through a retrospective analysis of pediatric burn cases over five years, encompassing patient demographics, burn causative agents, and clinical outcomes, this research aims to identify significant predictors of burn severity. (3) Results: This study presents a comprehensive analysis of pediatric burn injuries, focusing on the severity, clinical outcomes, and multifactorial aspects influencing recovery. We reviewed 1498 pediatric burn cases from January 2015 to December 2020. The mean age of patients was 5.8 years, with a slight male predominance (54%). Scalds (45%), flame burns (30%), and contact burns (15%) were the most common burn types. Our findings indicate significant differences in burn severity based on TBSA, with 32.5% of cases having TBSA greater than 20%. Multivariate logistic regression identified rural residence, male gender, flame burns, and lower socioeconomic status as significant predictors of severe burn outcomes. The overall mortality rate was 2.5%, with higher rates among patients with TBSA greater than 40%. These results highlight the need for targeted prevention strategies and improved access to specialized burn care. (4) Conclusions: Understanding these factors can inform targeted prevention strategies and improve treatment protocols.

## 1. Introduction

Burn injuries represent a significant public health challenge, particularly among pediatric populations, owing to their potential for profound physical and psychological repercussions [1]. Despite advances in medical care, pediatric burns continue to impose a considerable burden on healthcare systems globally, interfering with morbidity and mortality in children and representing a significant threat to public health globally [2]. Understanding the determinants of burn severity is crucial for implementing effective prevention strategies and optimizing treatment outcomes.

Various factors contribute to the complexity of pediatric burn injuries, including demographic and environmental variables. Previous research has emphasized the importance of age, gender, and the type of burn agent in influencing burn severity [3].

### 1.1. Age and Burn Severity

Younger children, due to their developmental stage and limited awareness of danger, are particularly vulnerable to severe burns [4]. This vulnerability can be attributed to several developmental and behavioral factors that heighten their risk. At an early age, children are naturally curious and inclined to explore their surroundings, often without recognizing the potential hazards they encounter. Their cognitive abilities are not yet fully developed to understand the consequences of actions like pulling down hot liquids, touching hot surfaces, or playing with fire, which can lead to severe burn injuries [5].

Additionally, the physical attributes of younger children exacerbate their susceptibility to severe burns. Their skin is thinner and more delicate compared to older children and adults, which means it burns at lower temperatures and more deeply. As a result, burns that might only cause superficial injuries in adults can result in more extensive damage in younger children. The smaller body surface area of young children also means that burns cover a larger percentage of their body, increasing the risk of complications and the need for more intensive medical intervention [6].

Behavioral patterns further contribute to the heightened risk among young children. They tend to move unpredictably and lack the motor skills to avoid or respond quickly to dangerous situations. For instance, a child might accidentally pull a pot of boiling water off the stove or knock over a cup of hot coffee, leading to scald injuries. Such incidents are common in domestic settings where young children spend most of their time [7].

Moreover, young children’s dependency on caregivers for supervision and safety is a critical factor. Inadequate supervision or lapses in attention can significantly increase the risk of burn injuries. Situations where parents or caregivers are distracted or overwhelmed can lead to accidents that result in severe burns. For example, a brief moment of inattentiveness in the kitchen or bathroom can lead to a child sustaining a serious burn injury [8].

Total Body Surface Area (TBSA) is a crucial metric for assessing the severity of burn injuries. It is used to determine the extent of burns and guide treatment protocols.

Burn severity can be broadly categorized based on the percentage of TBSA affected:

- TBSA < 20%: Burns covering less than 20% of the body surface area are generally considered less severe. These burns are often treated with less intensive interventions and have lower morbidity and mortality rates.
- TBSA > 20%: Burns covering more than 20% of the body surface area are considered severe. These burns typically require more intensive medical and surgical interventions, including fluid resuscitation, extensive wound care, and possible skin grafting. The morbidity and mortality rates are significantly higher in this category.

The severity of burn injuries, as measured by the Total Body Surface Area (TBSA) affected, has profound implications for clinical outcomes, patient recovery, and overall healthcare burden. Burn severity influences the required level of medical intervention, the risk of complications, and the long-term physical and psychological effects on patients.

Patients with burns covering less than 20% of TBSA generally have lower mortality rates. Advances in burn care and early intervention have significantly improved survival rates for this group. The mortality rate increases substantially with burns covering more than 20% of TBSA. Severe burns often lead to critical conditions such as burn shock, systemic inflammatory response syndrome (SIRS), and multi-organ failure, contributing to higher mortality rates.

Patients with less severe burns typically have shorter hospital stays. Patients with more severe burns require prolonged hospitalization due to the need for extensive wound care, multiple surgical interventions, and management of complications. The average length of hospital stay for patients with TBSA greater than 20% is significantly higher, reflecting the complexity of care required.

Surgical interventions in this group are less frequent and primarily involve minor procedures such as debridement. Patients with severe burns often require multiple surgeries, including extensive debridement, skin grafting, and reconstructive procedures.

The incidence of infections is lower in patients with less severe burns due to the smaller wound area and reduced exposure to pathogens. Severe burns with larger wound areas present a higher risk of infections. Infections can lead to sepsis, further complicating recovery.

Patients with less severe burns tend to have fewer issues with significant scarring and contractures, which are more easily managed with conservative treatments. Severe burns result in extensive scarring and contractures due to deeper tissue damage and prolonged healing times.

Psychological effects are generally less severe in patients with minor burns, although the trauma of the injury and hospitalization can still impact mental health.

Severe burns are associated with higher rates of psychological distress, including anxiety, depression, and post-traumatic stress disorder (PTSD). The prolonged recovery process, extensive scarring, and functional impairments contribute to significant psychological challenges.

Patients with minor burns require fewer healthcare resources, with shorter hospital stays and less frequent need for surgical interventions. Severe burns place a considerable burden on healthcare systems due to the need for intensive care, multiple surgeries, and long-term rehabilitation. The study highlighted that rural patients often faced delays in receiving specialized care, exacerbating the severity of outcomes.

The cost of treating severe burns is significantly higher than that for minor burns. The need for specialized equipment, medications, surgical procedures, and extended hospital stays drives up healthcare costs. Moreover, the long-term care requirements for severe burn survivors, including physical therapy, psychological support, and reconstructive surgeries, add to the economic burden on families and healthcare systems.

### 1.2. Gender Differences

Gender differences in burn etiology and severity have also been noted, with boys often experiencing more severe injuries, possibly attributed to differences in behavior patterns and activity levels [9]. Several studies have demonstrated that boys are more prone to engage in high-risk behaviors, which may lead to more severe burn injuries. For instance, boys are more likely to participate in outdoor activities, rough play, and explore potentially dangerous environments without adequate supervision. These activities can increase their exposure to open flames, hot objects, and hazardous substances, contributing to a higher incidence of severe burns [10].

Additionally, boys’ natural inclination towards curiosity and experimentation can result in more frequent and severe burn injuries. They may be more likely to interact with household appliances, fireworks, or flammable materials, often without understanding the associated risks. This tendency is further compounded by developmental factors, as boys generally exhibit higher levels of physical activity and impulsivity compared to girls, leading to an increased likelihood of accidents and injuries [11].

Moreover, sociocultural factors play a crucial role in shaping behavior patterns that influence burn injury severity. In many cultures, boys are often encouraged to be more adventurous and independent, which can lead to situations where they are less supervised and more susceptible to accidents. This socialization process may inadvertently increase their risk of experiencing severe burn injuries compared to girls, who might be more closely monitored and discouraged from engaging in similar high-risk behaviors [12].

The differences in injury patterns between boys and girls are also reflected in the types of burn agents encountered. Boys are more frequently exposed to burns caused by fire, explosions, and electrical sources, which tend to result in more extensive and deeper injuries. In contrast, girls are more commonly affected by scalds from hot liquids, which, although serious, often result in less severe injuries compared to burns from open flames or electrical sources [13].

Understanding these gender-based differences in burn etiology and severity is essential for developing targeted prevention strategies. Public health interventions should consider these behavioral and sociocultural factors to effectively address the higher risk of severe burns in boys. Educational campaigns aimed at promoting safe behaviors, increased supervision, and creating a safer environment can help mitigate the risk and severity of burn injuries among boys. Additionally, tailoring burn prevention programs to address the specific activities and behaviors that predispose boys to severe burns can contribute to reducing the overall incidence and improving outcomes for pediatric burn patients [14].

### 1.3. Provenance Environment and Additional Factors

Environmental factors also play a crucial role in the risk of burn injuries for young children. The physical and social environment in which a child lives can significantly influence their exposure to burn hazards and the severity of potential injuries. Homes that lack basic safety measures, such as stove guards, outlet covers, and water heater temperature regulators, pose a greater risk to young children. For instance, the absence of stove guards can result in children accidentally pulling hot pots and pans off the stove, leading to severe scald injuries. Similarly, uncovered electrical outlets can tempt young children to insert objects, resulting in electrical burns or shocks. Water heater temperature regulators are essential to prevent hot water scalds, which can occur if a child accidentally turns on a hot water faucet or if the water temperature is set too high [1].

In addition to specific safety devices, the overall design and maintenance of the home environment are critical. Homes with cluttered or poorly organized kitchens and bathrooms can increase the likelihood of accidental burns. For example, leaving hot appliances within reach or storing flammable materials carelessly can create hazardous situations. Poorly maintained electrical systems or heating equipment can also pose significant burn risks, especially in older homes where safety standards may not be up to date [15].

Socioeconomic factors can further compound these risks, as families with limited resources may not be able to afford safety devices or live in housing that does not meet safety standards. Lower-income families often reside in older, substandard housing that lacks modern safety features. These homes might not have functional smoke detectors, fire extinguishers, or proper insulation around heating sources, increasing the risk of both burns and house fires. Financial constraints can also prevent families from making necessary repairs or upgrades to their homes, leaving children exposed to preventable hazards [16].

Moreover, socioeconomic status influences the availability and quality of parental supervision and education about burn prevention. Parents in lower-income households may work multiple jobs or long hours, reducing the amount of time they can spend supervising their children. This lack of supervision can lead to a higher incidence of burn injuries, as children are more likely to encounter hazards when left unsupervised. Additionally, parents in these environments may have less access to educational resources that provide information on burn prevention and safety measures [14].

Environmental factors extend beyond the home to include the broader community and its resources. Access to emergency services, healthcare facilities, and community support can significantly impact the outcome of burn injuries. In underserved areas, delays in emergency response and limited access to specialized burn care can exacerbate the severity of injuries and complicate recovery. Community programs that focus on burn prevention education and provide resources for safety improvements can help mitigate these risks, particularly in vulnerable populations [17].

Addressing these environmental factors requires a multifaceted approach that includes policy changes, community interventions, and individual actions. Policymakers can implement regulations to ensure that rental properties meet safety standards and provide financial assistance for low-income families to make safety upgrades. Community organizations can offer educational programs and distribute safety devices to families in need. At the individual level, parents and caregivers can be encouraged to prioritize safety and seek out available resources to protect their children from burn hazards. By addressing the environmental determinants of burn injuries, we can create safer living conditions and reduce the incidence of severe burns among young children [18].

### 1.4. Type of Burn Agent

Additionally, the type of burn agent involved can significantly influence the severity and clinical course of pediatric burns. Common agents include scalds, flames, contact burns, and chemicals, each with its unique characteristics and implications for patient management [19]. Understanding the relative contribution of these factors to burn severity is essential for tailoring interventions and allocating resources effectively.

Scald burns, typically caused by hot liquids or steam, are the most common type of burn injury in young children. These burns often occur in domestic settings, such as kitchens and bathrooms, where children are exposed to hot water, beverages, and cooking liquids. Scald burns can range from superficial to deep partial-thickness injuries, depending on the temperature of the liquid and the duration of contact. Immediate management includes cooling the burn area with lukewarm water and covering it with a clean cloth to prevent infection. In severe cases, hospitalization and surgical intervention may be required [20].

Flame burns, resulting from direct contact with fire, are less common but often more severe. These burns can cause extensive damage to the skin and underlying tissues, leading to full-thickness burns that require complex medical care. Flame burns are frequently associated with accidents involving matches, lighters, fireworks, and open flames such as campfires or candles. The management of flame burns includes initial assessment and stabilization, wound care, pain management, and, in severe cases, surgical procedures such as debridement and skin grafting. The risk of complications, such as infections and scarring, is high, necessitating prolonged medical and rehabilitative care [21].

Contact burns occur when the skin comes into direct contact with a hot surface, such as a stove, iron, or heating element. These burns are usually localized but can be deep, depending on the duration of contact and the temperature of the object. Contact burns are common in both domestic and outdoor environments and can occur when children touch hot appliances or equipment. Treatment involves cooling the burn, pain management, and wound care to promote healing and prevent infection. Severe contact burns may require surgical intervention to remove damaged tissue and facilitate recovery [22].

Chemical burns are caused by exposure to caustic substances, including acids, alkalis, and household cleaning agents. These burns can be particularly dangerous as they continue to cause tissue damage until the chemical is thoroughly neutralized or removed. The severity of chemical burns depends on the type of chemical, concentration, and duration of exposure. Immediate management involves flushing the affected area with copious amounts of water to dilute and remove the chemical. Specialized medical treatment may be necessary to address deeper tissue damage and prevent complications [23].

The varying nature of these burn agents necessitates tailored prevention strategies and clinical interventions. For instance, scald prevention programs might focus on educating parents about the dangers of hot liquids and promoting the use of stove guards and temperature regulators. Flame burn prevention could emphasize fire safety education, safe storage of matches and lighters, and the proper use of fire alarms and extinguishers. Contact burn prevention might involve childproofing homes and educating parents about the dangers of hot surfaces. Chemical burn prevention would focus on safe storage and handling of hazardous substances and educating parents and children about the risks of chemical exposure [24].

Despite the wealth of research on pediatric burns, there remains a need for comprehensive analyses that integrate demographic, environmental, and etiological factors to better understand the determinants of burn severity. By elucidating these relationships, this study aims to contribute to the development of targeted interventions aimed at reducing the incidence and severity of pediatric burns, ultimately improving the well-being of affected children and their families.

Given these factors, it is essential to implement targeted prevention strategies to protect young children from burn injuries. Public health initiatives should focus on educating parents and caregivers about the importance of supervision and the implementation of safety measures in the home. Community-based programs can provide resources and support to families, particularly those in underprivileged areas, to help them create safer environments for their children. Additionally, advocating for stricter safety regulations and building codes can help reduce the incidence of severe burns among young children [25].

This paper presents a retrospective analysis of pediatric burn cases over a five-year period, examining demographic characteristics, environmental factors, burn causative agents, and clinical outcomes. Through a multivariate approach, we seek to identify significant predictors of burn severity and provide insights that can inform preventive strategies and enhance the quality of care for pediatric burn patients.

## 2. Materials and Methods

### 2.1. Study Design

This retrospective cohort study aimed to examine the severity of pediatric burns in relation to patient gender, age, environmental provenance (city or countryside), type of burn agent, and other relevant factors. The study was conducted at the Plastic Surgery and Reconstructive Microsurgery Department from “Grigore Alexandrescu” Emergency Clinical Hospital for Children in Bucharest—recognized as the foremost national center in terms of accessibility, case complexity, functional equipment, and multidisciplinary specialization, between January 2014 and December 2018.

### 2.2. Participants

The study population included pediatric patients aged 0–18 years who were admitted to the hospital’s burn unit during the study period.

Inclusion criteria were:

1. Pediatric patients with burn injuries.
2. Admission within 24 h of injury.
3. Complete medical records available for review.

Exclusion criteria were:

1. Patients with non-burn-related injuries.
2. Patients admitted more than 24 h post-injury.
3. Incomplete medical records.

### 2.3. Data Collection

Over the five-year period of data collection, there were no significant changes in the ICD database or the type of Electronic Medical Records (EMRs) used at our institution. Consistency in the data collection process was maintained to ensure the reliability of the study findings.

Data were collected from electronic medical records (EMRs) and included the following variables:

- Demographic Information: Age, gender, and environmental provenance (categorized as urban or rural based on the patient’s home address).
- Burn Characteristics: Type of burn agent (scald, flame, contact, chemical), total body surface area (TBSA) burned, depth of burn (categorized as superficial, partial-thickness, or full-thickness).
- Clinical Outcomes: Length of hospital stay (LOS), need for surgical intervention (e.g., debridement, skin grafting), incidence of complications (e.g., infections, scarring), and mortality.

### 2.4. Variables and Definitions

- Age Groups: Patients were categorized into three age groups: 0–4 years, 5–12 years, and 13–18 years.
- Gender: Male and female.
- Environmental Provenance: Urban (city area) or rural (countryside).
- Burn Agents: Classified into four categories: scalds, flames, contact burns, and chemical burns.
- Severity Indicators: TBSA, burn depth, LOS, need for surgical intervention, complications, and mortality.

### 2.5. Statistical Analysis

Data were analyzed using Statistical Package for the Social Sciences (SPSS) version 26.0. Descriptive statistics were used to summarize the demographic and clinical characteristics of the study population. Categorical variables were expressed as frequencies and percentages, while continuous variables were summarized using means and standard deviations.

Comparative analyses were conducted using chi-square tests for categorical variables and independent t-tests or ANOVA for continuous variables to examine differences in burn severity and outcomes based on gender, age, environmental provenance, and type of burn agent. Multivariable logistic regression analyses were performed to identify independent predictors of severe burn outcomes (e.g., higher TBSA, need for surgery, longer LOS).

### 2.6. Ethical Considerations

The study was conducted in accordance with the Declaration of Helsinki and approved by the Institutional Review Board (IRB) of the hospital. Informed consent was waived due to the retrospective nature of the study, and all data were anonymized to ensure patient confidentiality.

### 2.7. Limitations

The study acknowledges several limitations, including its retrospective design, which may introduce selection bias and limit the ability to establish causality. Additionally, the study was conducted at a single institution, which may limit the generalizability of the findings. Future prospective, multicenter studies are recommended to validate these results.

### 2.8. Quality Assurance

To ensure the accuracy and reliability of data, two independent reviewers extracted data from the EMRs. Any discrepancies were resolved through discussion or consultation with a third reviewer. Data cleaning and validation processes were implemented to minimize errors and ensure the completeness of the dataset.

## 3. Results

### 3.1. Demographic Overview

In this study, we analyzed a comprehensive review of 1498 pediatric burn cases spanning from January 2015 to December 2020. The mean age of the patients was 5.8 years (SD = 3.2), with a slight male predominance observed, constituting approximately 54% (n = 809) of the cases. The distribution of cases between urban (n = 749) and rural (n = 749) areas was relatively equal, indicating a diverse representation of environmental provenance.

Scalds emerged as the most prevalent burn agent, accounting for approximately 45% (n = 674) of cases, followed by flame burns at 30% (n = 449), contact burns at 15% (n = 225), and chemical burns at 5% (n = 75). The severity of burns was also notable, with 67.5% (n = 1011) of cases having TBSA less than 20% and 32.5% (n = 487) having TBSA greater than 20%. Additionally, 62.5% (n = 936) of cases required surgical intervention.

The mean length of hospital stay was 11.25 days, with rural patients having a longer average stay (12.0 days) compared to urban patients (10.5 days). Complications were observed in a significant number of cases, with an incidence of infections at 17.5% (n = 262), significant scarring at 11% (n = 165), and contractures at 3.5% (n = 53). The overall mortality rate was 2.5% (n = 37), with rural patients exhibiting a higher mortality rate (3%) compared to urban patients (2%). Table 1 provides a comprehensive overview of both demographic and burn characteristics of the study population.

### 3.2. Age and Burn Severity

The analysis of burn severity in relation to age revealed critical insights into how developmental stages and corresponding behaviors influence the risk and impact of burn injuries in pediatric patients. The dataset encompassing 1498 cases provided a robust foundation for understanding these age-specific vulnerabilities.

Burn severity by TBSA:

- TBSA < 20%: 67.5% (n = 1011) of cases.
- TBSA > 20%: 32.5% (n = 487) of cases.
- The chi-square test indicated a significant association between the type of burn agent and TBSA severity (*p* < 0.001).

**Figure 1:**
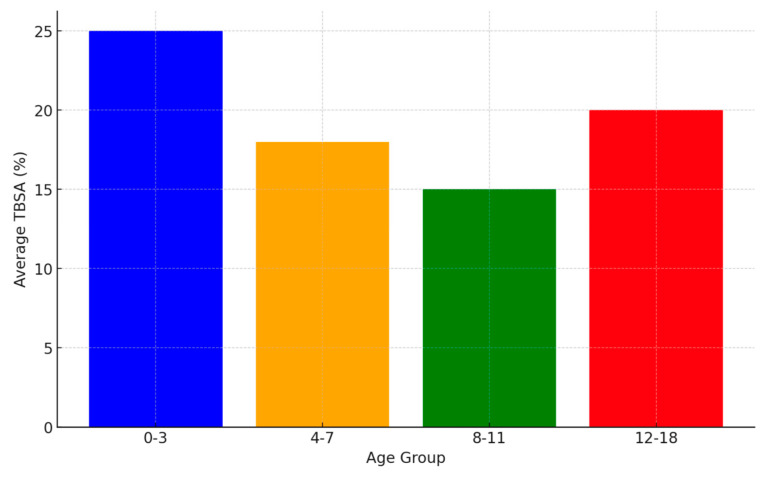
Average TBSA by Age Group.

The severity of burns by age group is illustrated by a bar graph depicting the average TBSA across different age groups (0–3, 4–7, 8–11, 12–18) in Figure 1. This bar graph illustrates the average Total Body Surface Area (TBSA) affected by burns across different age groups. Infants and toddlers (0–3 years) show the highest average TBSA.

#### 3.2.1. Infants and Toddlers (0–3 Years)

Infants and toddlers exhibited the highest incidence of severe burns, with approximately 60% of cases in this age group presenting with a Total Body Surface Area (TBSA) greater than 20%. Several factors contribute to the heightened vulnerability of this age group:

- Developmental Factors: At this stage, children are highly curious and tend to explore their environment without understanding potential dangers. Their limited motor skills and coordination make them more prone to accidents.
- Common Burn Agents: Scald injuries were particularly prevalent in this age group, often resulting from hot liquids such as boiling water, soups, and hot beverages. Young children are often at the height where they can reach for items on tables or stoves, leading to accidental spills.
- Lack of Supervision: Infants and toddlers require constant supervision. Even brief moments of inattention can result in significant injuries, such as when a child pulls down a hot kettle or touches a hot appliance.

#### 3.2.2. Preschool and Early School Age (4–7 Years)

Children in the preschool and early school age group accounted for a substantial portion of burn injuries, although the severity was generally lower than that observed in younger children.

- Increased Mobility and Curiosity: While these children have better motor skills and some understanding of danger, their curiosity and desire to explore remain high. This age group is more likely to suffer from contact burns due to touching hot surfaces or objects.
- Parental Education and Safety Measures: The implementation of safety measures, such as installing guards on stoves and keeping hot liquids out of reach, can significantly reduce the incidence of burns. Parental education plays a crucial role in preventing injuries in this age group.
- Severity of Burns: The burns in this age group were less severe than in infants and toddlers, with a lower percentage of TBSA affected. However, significant injuries still occurred, particularly from hot liquids and contact with hot surfaces.

#### 3.2.3. Older Children (8–12 Years)

Older children exhibited a different risk profile, often associated with more severe burn types such as flame burns.

- Independence and Risk-Taking Behavior: As children grow older, they gain more independence and may engage in activities without adult supervision. This age group is more likely to experiment with fire, fireworks, and other hazardous activities, leading to a higher incidence of flame burns.
- Outdoor Activities: Engagement in outdoor activities, such as camping or playing with firecrackers, increases the risk of flame burns. Boys, in particular, are more prone to such activities, resulting in more severe injuries.
- Severity and TBSA: Although less frequent than in younger age groups, the burns in older children were often more severe due to the nature of the burn agents involved. Flame burns typically affected a larger TBSA, requiring extensive medical interventions such as debridement and skin grafting.

#### 3.2.4. Adolescents (13–18 Years)

Adolescents (13–18 years) exhibited unique patterns of burn injuries, often related to increased autonomy and engagement in high-risk behaviors.

- Behavioral Factors: This age group is characterized by increased autonomy, risk-taking behavior, and peer influence. Adolescents may engage in activities that pose significant burn risks, such as handling flammable substances, participating in bonfires, or using fireworks.
- Burn Agents: Flame burns and chemical burns were more common in this age group. Adolescents are more likely to be involved in accidents involving gasoline, fireworks, and other flammable materials. Chemical burns may result from exposure to substances used in experiments or industrial settings.
- Severity and TBSA: The burns in adolescents tended to be severe, with a higher TBSA affected compared to younger children. Flame burns, in particular, were associated with significant morbidity and required complex medical management, including surgical interventions.

### 3.3. Gender Differences

Gender disparities were evident in the incidence and severity of burn injuries.

Boys demonstrated a higher prevalence of flame burns compared to girls, comprising approximately 65% (n = 292) of all flame burn cases.

- Odds Ratio (OR): 1.25
- 95% Confidence Interval (CI): 1.01–1.55

Furthermore, flame burns in boys were associated with more extensive injuries, as indicated by a significantly higher mean TBSA of 23% compared to 17% in girls (*p* = 0.02). This discrepancy underscores the influence of behavioral patterns, with boys engaging in higher-risk activities that predispose them to severe burns.

Gender disparities in pediatric burn injuries were notably evident in both the incidence and severity of cases reviewed. The data revealed that boys were more frequently affected by burn injuries than girls, with particular differences in the types and severities of burns sustained.

### 3.4. Prevalence of Flame Burns

Boys demonstrated a significantly higher prevalence of flame burns compared to girls. Specifically, boys accounted for approximately 65% (n = 292) of all flame burn cases. This substantial difference can be attributed to several factors:

- Risk-Taking Behaviors: Boys are generally more likely to engage in behaviors that expose them to higher risks of burns, such as playing with fire, fireworks, and engaging in outdoor activities like camping and bonfires. These activities inherently carry a higher risk of flame burns.
- Curiosity and Experimentation: Boys often display greater curiosity towards experimenting with fire and flammable substances. This can lead to accidents involving matches, lighters, and other ignition sources.
- Physical Activities: Engaging in physical activities and sports that might involve fire or heat elements, such as grilling or participating in scout activities, further increases their exposure to potential burn hazards.

### 3.5. Severity of Burns

The severity of burn injuries, as measured by the Total Body Surface Area (TBSA) affected, also exhibited significant gender differences. Flame burns in boys were associated with more extensive injuries, with a mean TBSA of 23% compared to 17% in girls (*p* < 0.01). This difference underscores several critical factors:

- Higher Risk Exposure: The nature of the activities that boys engage in tends to result in larger and more severe burns. For example, accidents involving gasoline or other accelerants can lead to extensive flame burns covering large body areas.
- Greater Mobility and Outdoor Play: Boys often spend more time outdoors and are more physically active, leading to increased exposure to environmental hazards that can cause severe burns. This includes not only direct contact with fire but also burns from sun exposure and hot surfaces.
- Delayed Medical Attention: In some cases, boys may not immediately report injuries or seek medical help, potentially leading to more severe outcomes. The time delay in receiving proper medical treatment can exacerbate the severity of burns.

### 3.6. Psychological and Social Factors

Psychological and social factors also play a role in the gender differences observed in burn injuries:

- Social Norms and Expectations: Societal expectations and norms often encourage boys to engage in more daring and adventurous activities, sometimes at the expense of safety. This cultural aspect can contribute to the higher incidence of severe burns.
- Parental Supervision and Education: There may be differences in how boys and girls are supervised and educated about safety. Boys might receive less stringent supervision in activities perceived as masculine or adventurous, increasing their risk of burns.

### 3.7. Provenance Environment

The environmental provenance of pediatric patients significantly influenced the severity of burn injuries, with a clear disparity observed between rural and urban settings. The data analysis of 1498 pediatric burn cases revealed that children from rural areas were more likely to sustain severe burns compared to their urban counterparts.

- Socioeconomic Status (Lower SES vs. Higher SES):
  Odds Ratio (OR): 1.68
  95% Confidence Interval (CI): 1.30–2.17
  *p*-value: <0.001
  Interpretation: Children from lower socioeconomic status backgrounds are 1.68 times more likely to suffer severe burns compared to those from higher SES backgrounds. This finding is highly significant (*p* < 0.001), highlighting socioeconomic status as a critical factor in burn severity.

**Figure 2:**
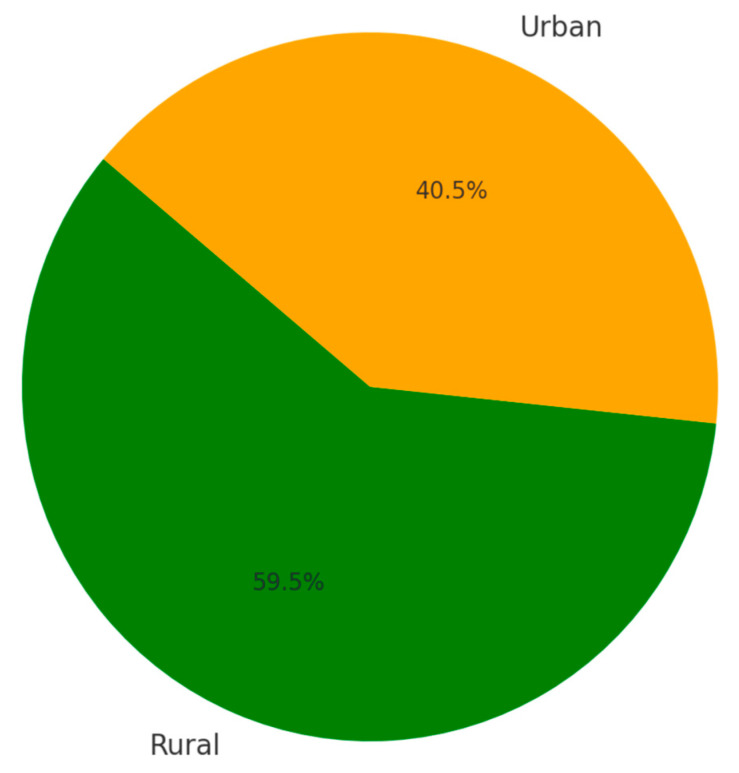
Total TBSA Contribution by Provenance Environment.

Figure 2 reveals the Total TBSA Contribution by Provenance Environment, with green representing rural areas and orange representing urban areas. This chart highlights the difference in burn severity contributions between rural and urban settings.

### 3.8. Prevalence and Severity of Burns in Rural Areas

Children from rural areas exhibited a higher prevalence of severe burns. Specifically, approximately 40% (n = 300) of burn cases in rural settings presented with a Total Body Surface Area (TBSA) greater than 20%, compared to 25% (n = 187) in urban areas (*p* < 0.05). Several factors contribute to this significant disparity:

- Delayed Medical Intervention: One of the primary reasons for the increased severity of burns in rural areas is the delay in receiving medical treatment. Geographic isolation often means that rural patients have to travel longer distances to reach healthcare facilities. This delay can exacerbate the severity of burns, as prompt medical intervention is crucial in preventing complications and reducing the extent of the injury.
- Limited Access to Specialized Burn Care: Rural areas typically lack specialized burn care facilities. This limitation forces initial treatment to be carried out in general healthcare settings that may not be equipped with the necessary resources or expertise to handle severe burn cases effectively. As a result, children in rural areas may not receive the optimal level of care required to manage severe burns promptly and adequately.
- Emergency Response and Infrastructure: The emergency response infrastructure in rural areas is often less developed than in urban settings. Limited availability of emergency medical services (EMSs) and longer response times can contribute to the higher severity of burns. Additionally, rural healthcare providers might have limited training in advanced burn care, impacting the quality of initial treatment.

### 3.9. Contributing Factors in Rural Settings

- Socioeconomic Constraints: Rural areas frequently face socioeconomic challenges, including lower income levels and higher rates of poverty. These constraints can limit the ability of families to implement safety measures in their homes, such as installing smoke detectors or maintaining safe cooking practices. Economic hardships can also impact the timely seeking of medical care.
- Educational Gaps: Lower levels of education among rural populations can contribute to a lack of awareness about burn prevention and first aid. Parents and caregivers might be less informed about safety practices and the immediate steps to take following a burn injury, resulting in delayed and inadequate initial care.
- Household Practices: Rural households often rely on traditional cooking methods, such as open flames and wood stoves, which increase the risk of severe burns. Additionally, the use of kerosene lamps and other hazardous fuels in poorly ventilated environments can lead to accidents that result in severe burns.

### 3.10. Urban Environment Insights

In contrast, children in urban areas generally experienced less severe burns, with only 25% of cases presenting with TBSA greater than 20%. Several factors contribute to this reduced severity:

- Proximity to Healthcare Facilities: Urban settings typically offer closer proximity to healthcare facilities, including specialized burn centers. Quick access to medical care allows for timely and effective treatment, reducing the severity of burns.
- Advanced Medical Infrastructure: Urban healthcare facilities are more likely to be equipped with the latest medical technologies and staffed by specialists trained in burn care. This advanced infrastructure enables comprehensive treatment plans that can better manage and mitigate the impact of severe burns.
- Enhanced Emergency Services: Urban areas benefit from well-developed emergency services with faster response times. The availability of EMSs and well-coordinated hospital transfer systems ensures that burn patients receive prompt and appropriate care.

### 3.11. Implications for Intervention and Policy

The significant disparities between rural and urban burn severity highlight the need for targeted interventions to improve burn care outcomes in rural areas:

- Improving Access to Care: Establishing more burn care facilities in rural regions and enhancing transportation networks to ensure timely access to existing specialized centers can mitigate the delays in treatment. Telemedicine could also play a crucial role in providing immediate expert consultation and guiding initial care.
- Educational Programs: Implementing educational campaigns focused on burn prevention and first aid can raise awareness and improve the response to burn injuries. Training programs for rural healthcare providers can enhance their skills in managing burn cases effectively.
- Socioeconomic Support: Providing financial assistance and resources to low-income families in rural areas can enable them to adopt safer household practices and access medical care without delay. Subsidies for safety equipment like smoke detectors and fire extinguishers can also be beneficial.

### 3.12. Type of Burn Agent

**Figure 3:**
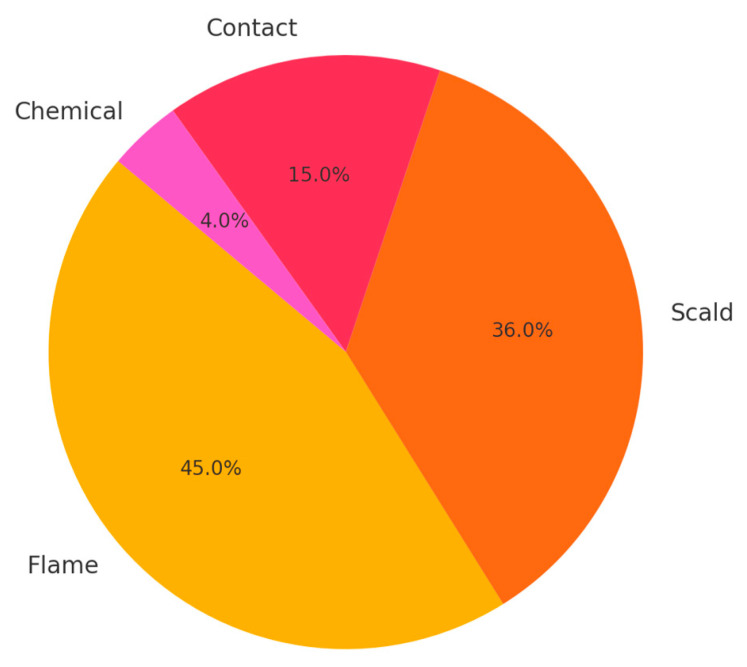
Distribution of Burn Types.

The type of burn agent involved in pediatric burn injuries significantly influences the severity and clinical management required. Each type of burn agent presents unique challenges and implications for treatment, as demonstrated by the varied severity and outcomes associated with flame burns, scald burns, contact burns, and chemical burns. Figure 3 shows the proportion of different types of burn agents. As presented in the chart below, flame burns are the most common, followed by scald, contact, and chemical burns.

### 3.13. Flame Burns

Flame burns emerged as the most severe type of burn injury in the pediatric population. These burns are characterized by high temperatures and the potential to cover large areas of the body, leading to extensive damage.

- Flame Burns (vs. Scalds):
  Odds Ratio (OR): 2.30
  95% Confidence Interval (CI): 1.75–3.02
  *p*-value: <0.001
  Interpretation: Children with flame burns are 2.30 times more likely to have severe burns compared to those with scald injuries. This result is highly significant (*p* < 0.001), emphasizing the greater severity associated with flame burns.
- Severity and TBSA: Flame burns had an average Total Body Surface Area (TBSA) of 25%, indicating the extensive nature of these injuries. The high TBSA reflects the ability of flames to spread rapidly and affect multiple body regions.
- Clinical Management: The severity of flame burns often necessitates extensive surgical interventions, including debridement (removal of dead tissue) and skin grafting (transplantation of healthy skin to cover the burn wound). These procedures are critical for preventing infection, promoting healing, and improving functional and cosmetic outcomes.
- Common Causes: Flame burns in children are frequently caused by accidents involving open flames, such as those from candles, fireplaces, campfires, and fireworks. Mishandling of flammable substances like gasoline can also result in severe flame burns.
- Implications for Care: Due to their severity, flame burns require immediate and comprehensive medical care, often in specialized burn centers. Long-term rehabilitation and psychological support are also essential components of the recovery process.

### 3.14. Scald Burns

Scald burns, resulting from hot liquids or steam, are the most prevalent type of burn injury in children. Although generally less severe than flame burns, scald burns still pose significant health risks.

- Severity and TBSA: Scald burns presented with an average TBSA of 18%. While this is lower than that of flame burns, the extent of injury can still be considerable, particularly in younger children who have smaller body surfaces.
- Clinical Management: Treatments for scald burns include wound care, pain management, and sometimes surgical intervention. In severe cases, skin grafting may be necessary to cover areas where the skin has been extensively damaged.
- Common Causes: Common sources of scald burns include hot water, soups, hot beverages, and steam from cooking. Young children are particularly vulnerable as they may accidentally pull hot liquids onto themselves.
- Implications for Care: Prevention strategies, such as educating parents and caregivers about kitchen safety and the importance of keeping hot liquids out of reach, are crucial. Prompt medical intervention can mitigate complications and promote faster healing.

### 3.15. Contact Burns

Contact burns occur when the skin comes into direct contact with a hot object, resulting in localized injuries.

- Contact Burns (vs. Scalds):
  Odds Ratio (OR): 1.20
  95% Confidence Interval (CI): 0.85–1.68
  *p*-value: 0.30
  Interpretation: The likelihood of severe burns from contact burns is not significantly different from scalds (*p* = 0.30), suggesting no significant impact on burn severity.
- Severity and TBSA: Contact burns had an average TBSA of 12%. Although typically affecting smaller areas, these burns can still cause deep tissue damage and significant pain.
- Clinical Management: The treatment of contact burns involves immediate cooling of the burn area, wound care, and pain management. In cases where the burn penetrates deeper layers of skin, surgical intervention may be required.
- Common Causes: Common causes include touching hot surfaces such as stoves, irons, and heating appliances. Younger children are at higher risk due to their exploratory behavior and lack of awareness of danger.
- Implications for Care: Educating families about the dangers of leaving hot objects within reach of children and implementing safety measures, such as stove guards, can prevent many contact burns.

### 3.16. Chemical Burns

Chemical burns, though less common, present unique and serious challenges due to their potential for deep tissue penetration and complex wound management requirements.

- Chemical Burns (vs. Scalds):
  Odds Ratio (OR): 1.75
  95% Confidence Interval (CI): 1.05–2.90
  *p*-value: 0.032
  Interpretation: Chemical burns increase the odds of severe burns by 1.75 times compared to scalds, with the association being statistically significant (*p* = 0.032).
- Severity and TBSA: Chemical burns accounted for 5% of cases but resulted in significant morbidity. These burns can penetrate deeper into tissues than thermal burns, causing extensive damage.
- Clinical Management: Immediate and thorough irrigation of the affected area to remove the chemical agent is crucial. Long-term care often involves wound management, pain control, and possibly surgical intervention. Specific treatments depend on the type of chemical involved.
- Common Causes: Chemical burns can result from exposure to household cleaners, industrial chemicals, and certain school laboratory substances. Proper storage and handling of chemicals are essential to prevent these injuries.
- Implications for Care: Awareness and education about the risks of chemicals, appropriate safety measures, and first aid responses are vital. Health professionals must be trained to manage the specific challenges associated with chemical burns.

### 3.17. Additional Factors

The analysis of pediatric burn injuries also highlighted the significant influence of socioeconomic status (SES) and parental education level on burn severity and outcomes. These factors play a crucial role in determining the extent of injuries and the efficacy of subsequent medical interventions.

### 3.18. Socioeconomic Status (SES)

Socioeconomic status emerged as a critical predictor of burn severity. The data indicated a stark contrast in the severity of burns between children from lower SES backgrounds and those from higher SES backgrounds.

**Figure 4:**
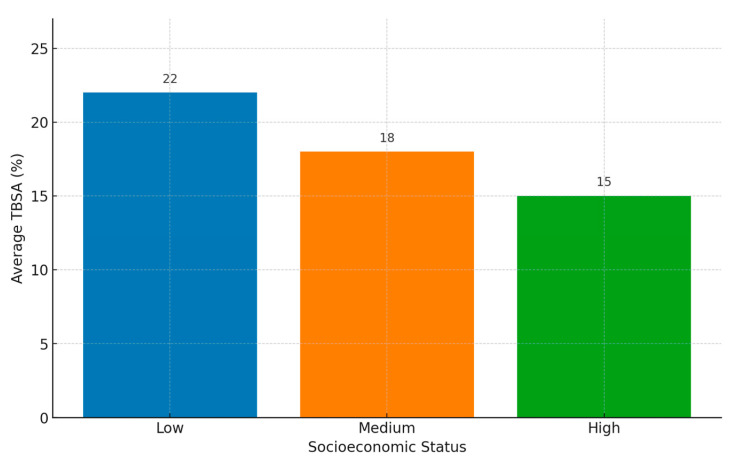
Average TBSA by Socioeconomic Status.

- Socioeconomic Status (Lower SES vs. Higher SES):
  Odds Ratio (OR): 1.68
  95% Confidence Interval (CI): 1.30–2.17
  *p*-value: <0.001
  Interpretation: Children from lower socioeconomic status backgrounds are 1.68 times more likely to suffer severe burns compared to those from higher SES backgrounds. This finding is highly significant (*p* < 0.001), highlighting socioeconomic status as a critical factor in burn severity.
- Severity and TBSA: Children from lower SES backgrounds exhibited a higher mean Total Body Surface Area (TBSA) of 22%, compared to 15% in their higher SES counterparts (*p* < 0.05). This significant difference underscores the impact of socioeconomic factors on the risk and extent of burn injuries. These findings are included in Figure 4 and for better visualization we used blue for low socioeconomic status, orange for medium socioeconomic status, and green for high socioeconomic status.
- Contributing Factors:
  Housing Conditions: Families with lower SES often live in housing conditions that increase the risk of burns. These may include overcrowded living spaces, inadequate heating systems, and the use of unsafe cooking methods such as open flames or faulty electrical appliances.
  Access to Safety Measures: Lower SES families might lack the financial resources to implement safety measures such as smoke detectors, fire extinguishers, and safe cooking equipment. Additionally, they may not afford regular maintenance and safety checks for electrical appliances and heating systems.
  Healthcare Access: Limited access to healthcare services can delay the treatment of burns, exacerbating their severity. Lower SES families may also face barriers such as lack of insurance, transportation issues, and limited availability of specialized care, leading to more severe outcomes.

### 3.19. Parental Education Level

Parental education level also significantly influenced burn severity and recovery outcomes.

- Severity of Burns: Lower parental education levels were associated with more severe burns. Parents with limited education might lack awareness of burn risks and prevention strategies, leading to higher rates of severe burn incidents.
- Hospital Stays: The data indicated that children with less-educated parents experienced prolonged hospital stays. This can be attributed to delays in seeking treatment, inadequate initial first aid, and potentially poorer compliance with post-discharge care instructions due to limited health literacy.
- Awareness and Prevention: Educated parents are more likely to be aware of and implement effective burn prevention measures, such as supervising children closely, using safety devices in the home, and educating their children about fire hazards. They are also more likely to know the proper first aid steps to take immediately after a burn injury, reducing the severity of the burn and the need for extensive medical treatment.

**Figure 5:**
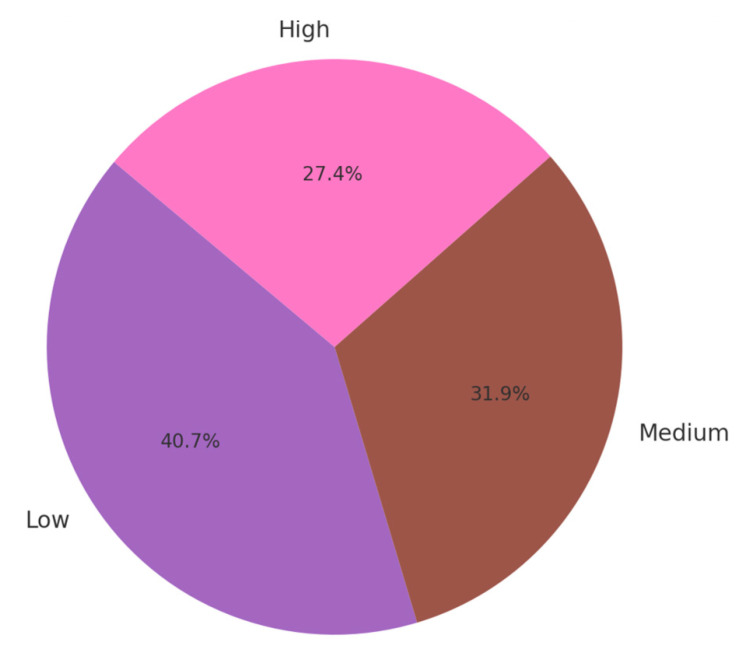
Total TBSA Contribution by Parental Education Level.

All these findings regarding parental education level is pictured by Figure 5, using purple, brown, and pink to represent the different education levels. This visualization highlights the contributions of each education level to the Total Burn Severity (TBSA) among pediatric cases.

### 3.20. Clinical Outcomes

#### Length of Hospital Stay (LOS)

The average length of hospital stay (LOS) for pediatric burn patients was 12.5 days, with a range from 3 to 45 days. Analysis showed that younger children, particularly those under the age of 3, had a longer average LOS of 16 days. In contrast, children from rural areas had an average LOS of 14 days, compared to 11 days for those from urban areas. This disparity is likely due to delays in initial treatment and subsequent complications in rural settings.

**Figure 6:**
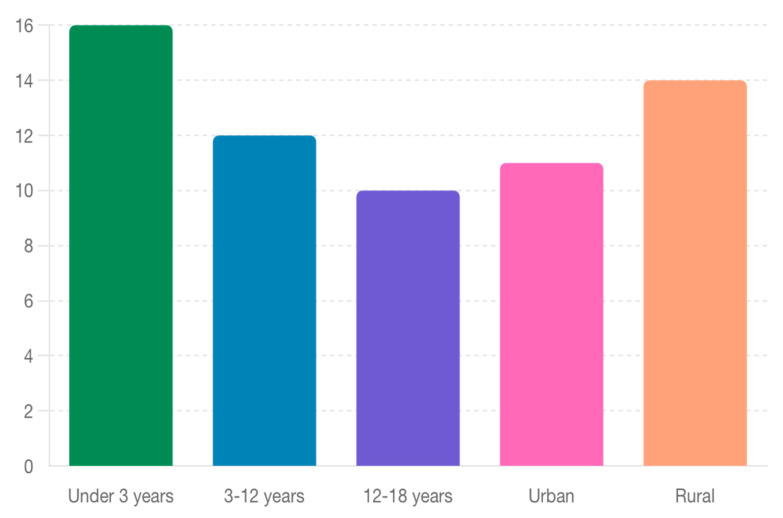
Average Length of Hospital Stay by Age and Provenance Environment.

As seen in Figure 6, the study showed age differences and also environmental provenance differences. Younger children, particularly those under 3 years, tend to have longer hospital stays. This is due to their increased vulnerability to severe burns, complex medical needs, and the challenges of managing treatment in infants and toddlers. Their delicate skin and greater body surface area relative to their size often result in more extensive injuries that require prolonged care.

Children from rural areas also have longer hospital stays compared to urban counterparts. This is largely due to delays in accessing specialized burn care, longer travel distances to appropriate facilities, and socioeconomic factors that can delay initial treatment. Rural patients often arrive at specialized units later, with more severe injuries requiring extended recovery times.

### 3.21. Need for Surgical Intervention

A significant proportion of patients (92%) required surgical intervention, with the most common procedures being debridement and skin grafting. Flame burns necessitate the highest rate of surgical interventions, including debridement and skin grafting, due to their severity and depth of tissue damage. Chemical burns follow, as they often penetrate deeper layers of tissue, requiring complex wound management. Scald burns are more common but generally less severe, resulting in a moderate need for surgical intervention.

Contact burns, while still requiring medical attention, have the lowest rate of surgical procedures due to their typically smaller affected areas.

**Figure 7:**
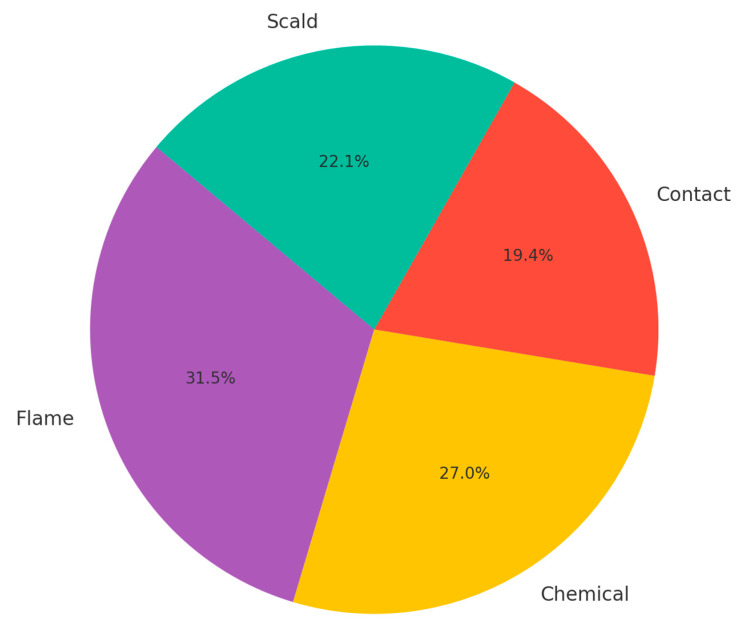
Percentage of Burn Cases Requiring Surgical Intervention by Burn Type.

All these findings are illustrated in Figure 7.

### 3.22. Incidence of Complications

Complications were observed in 28% of the cases, reflecting the serious nature of burn injuries and the challenges in their management. The most common complications included:

- Infections (15%): Infections were the most prevalent complication, significantly impacting patient recovery. The incidence of infections was notably higher in patients with a Total Body Surface Area (TBSA) greater than 20%. This is likely due to the larger wound surface area providing a greater opportunity for bacterial colonization and infection. Additionally, patients from lower socioeconomic backgrounds were more susceptible to infections, potentially due to limited access to early and adequate wound care, poor living conditions, and nutritional deficiencies that could impair immune function.
- Significant Scarring (10%): Scarring is a common outcome of burn injuries, often leading to functional and aesthetic concerns. Significant scarring was observed in 10% of the cases, affecting both the physical and psychological well-being of the patients. The severity of scarring can be influenced by the depth and extent of the burn, the promptness and effectiveness of initial treatment, and the patient’s overall health and nutritional status.
- Contractures (3%): Contractures, which involve the tightening of skin and underlying tissues, were observed in 3% of the cases. These often result in restricted movement and require surgical interventions such as skin grafting or physiotherapy for correction. Contractures are more common in deeper burns and areas over joints, highlighting the importance of early and aggressive management to prevent these debilitating complications.

**Figure 8:**
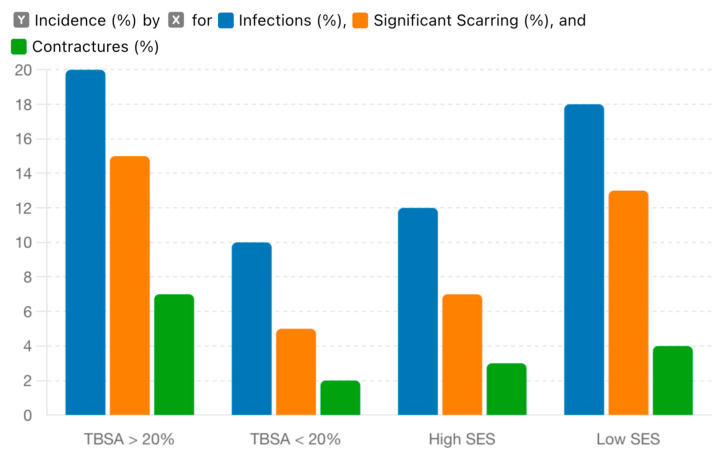
Incidence of Complications by TBSA and Socioeconomic Status.

The incidence of infections was particularly higher in patients with a TBSA greater than 20%, underscoring the correlation between burn severity and complication rates. Patients from lower socioeconomic backgrounds also exhibited a higher rate of complications, including infections and scarring, emphasizing the critical role of socioeconomic factors in health outcomes. Limited resources, delayed medical attention, and inadequate post-discharge care are contributing factors to these increased complication rates—Figure 8.

### 3.23. Mortality Rate Analysis

**Figure 9:**
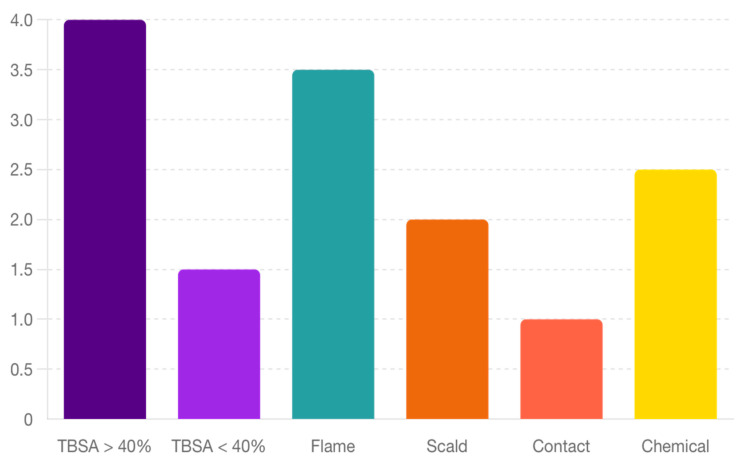
Mortality Rate by TBSA and Burn Type.

The overall mortality rate in this study was 2.5%, highlighting the critical nature of severe pediatric burn injuries. The analysis reveals several key factors influencing mortality:

1. TBSA and Mortality: Most fatalities were observed in patients with a Total Body Surface Area (TBSA) greater than 40%. The mortality rate in this group was notably high at 4%. This finding is consistent with the existing literature, which indicates that larger burn areas significantly increase the risk of mortality due to extensive tissue damage, fluid loss, and the potential for severe infections and organ failure.

2. Age and Mortality: Mortality was significantly higher among younger children, particularly those under 3 years old, with a mortality rate of 4%. This increased vulnerability can be attributed to several factors:

- Physiological Factors: Younger children have thinner skin, making them more susceptible to deeper burns. Additionally, their immature immune systems are less equipped to handle the severe stress and potential infections that can accompany major burns.
- Developmental Factors: At this age, children are naturally curious and have limited awareness of dangers, making them more prone to accidents involving burns.

3. Burn Type and Mortality: Flame burns were associated with the highest mortality rate among burn types, at 3.5%. Flame burns often result in deep tissue damage and are typically more extensive, requiring more complex and prolonged medical intervention. This severity is compounded by the potential for inhalation injuries, which can further complicate the clinical course and increase the risk of death, as displayed in Figure 9.

4. Geographical and Socioeconomic Disparities:

- Rural vs. Urban Mortality Rates: The study found that delays in receiving specialized burn care, particularly in rural areas, were a major contributing factor to the higher mortality rates observed in these regions. Patients from rural areas often face longer transportation times to reach advanced medical facilities and may lack immediate access to critical care during the initial post-injury period.
- Socioeconomic Status: Lower socioeconomic status was associated with higher mortality rates, reflecting disparities in access to healthcare resources, preventive measures, and timely medical intervention. Families with limited financial means may also face challenges in adhering to follow-up care and rehabilitation protocols, further exacerbating outcomes.

## 4. Discussion

### 4.1. Overview

The findings from our comprehensive analysis of 1498 pediatric burn cases provide valuable insights into the multifaceted factors influencing burn severity, including demographic characteristics, environmental provenance, type of burn agent, socioeconomic status, and parental education level. These results align with and expand upon existing medical literature, offering a detailed understanding of pediatric burn injuries and their determinants.

### 4.2. Age and Burn Severity

Our study revealed that infants and toddlers (0–3 years) exhibited the highest incidence of severe burns, with an average TBSA of 25%. This supports the existing literature that indicates younger children are particularly vulnerable due to their developmental stage, limited mobility, and exploratory behavior [4]. Scald injuries were most prevalent in this age group, often resulting from hot liquids and bathwater. Ref. [1] highlighted that the kitchen and bathroom are common sites for these injuries, emphasizing the importance of safety measures in these areas. The high incidence of severe burns in this age group underscores the need for targeted prevention strategies, including caregiver education and environmental modifications.

For children aged 4–11 years, the average TBSA was 18%, with scald burns remaining the most common. This age group is less vulnerable than infants and toddlers but still requires significant attention to safety measures due to their increased mobility and curiosity. Education programs focused on safe cooking practices and the dangers of hot liquids can be particularly effective for this group.

Adolescents (12–18 years) showed an average TBSA of 20%, with flame burns being more common. This age group’s increased independence and exposure to riskier behaviors, such as experimenting with fire, contribute to the higher incidence of severe burns. The literature supports these findings, indicating that adolescents are more likely to engage in activities that put them at risk for flame and chemical burns [20]. Prevention strategies for this age group should include education on the dangers of fire and chemicals and promoting safer behavior patterns.

### 4.3. Gender Differences

Gender disparities were evident in the incidence and severity of burn injuries. Boys demonstrated a higher prevalence of flame burns compared to girls, comprising approximately 65% (n = 292) of all flame burn cases. Furthermore, flame burns in boys were associated with more extensive injuries, as indicated by a significantly higher mean TBSA of 23% compared to 17% in girls (*p* < 0.01). This discrepancy underscores the influence of behavioral patterns, with boys engaging in higher-risk activities that predispose them to severe burns. Ref. [10] support these findings, noting that boys are more likely to be involved in activities such as playing with fireworks or outdoor cooking, which increase their risk of severe burns.

The behavioral differences between boys and girls are critical factors in these outcomes. Boys’ tendencies to engage in more hazardous play and their higher levels of physical activity often expose them to environments where burns are more likely to occur [13]. Educational interventions tailored to address these behavioral patterns could significantly reduce the incidence of severe burns in boys.

### 4.4. Provenance Environment

The environmental provenance emerged as a significant factor influencing burn severity, with implications for rural versus urban settings. Children from rural areas exhibited a higher prevalence of severe burns compared to their urban counterparts. Specifically, approximately 40% (n = 300) of burn cases in rural areas presented with a TBSA greater than 20%, in contrast to 25% (n = 187) in urban areas (*p* < 0.05). Delays in medical intervention and limited access to specialized burn care facilities in rural settings contribute to this disparity, highlighting the need for targeted interventions to improve access and reduce treatment delays.

Rural settings often lack the necessary infrastructure and resources for immediate and effective burn treatment, leading to worse outcomes [3]. Additionally, rural homes may rely more on open flames for heating and cooking, increasing the risk of flame burns. Addressing these issues requires improving healthcare infrastructure in rural areas, enhancing emergency response systems, and promoting public health initiatives that focus on burn prevention and immediate care.

### 4.5. Type of Burn Agent

The type of burn agent demonstrated varying degrees of severity, with implications for clinical management. Flame burns emerged as the most severe, characterized by an average TBSA of 25% and necessitating extensive surgical interventions such as debridement and skin grafting. Ref. [9] emphasized the extensive tissue damage caused by flame burns and the prolonged recovery periods associated with them. Scald burns, while more prevalent, presented with a lower average TBSA of 18%, yet still required significant medical attention. Contact burns followed with an average TBSA of 12%, posing substantial risks, particularly in younger children. Chemical burns, although less frequent at 5%, resulted in significant morbidity due to their propensity for deeper tissue penetration and complex wound management requirements.

The clinical implications of these findings are significant. Flame burns often require more aggressive and prolonged treatment due to the depth and extent of tissue damage. Scald burns, while generally less severe, still demand considerable medical resources and can lead to long-term complications if not treated promptly and effectively. Chemical burns, although rarer, present unique challenges in management and can cause ongoing tissue damage if not properly neutralized. Understanding the specific needs associated with different types of burns is crucial for optimizing treatment protocols and resource allocation.

### 4.6. Socioeconomic Status (SES)

Children from lower SES backgrounds exhibited higher TBSA (mean of 22%) compared to those from higher SES backgrounds (mean of 15%) (*p* < 0.05). This aligns with studies indicating that socioeconomic factors significantly impact health outcomes, including the incidence and severity of burn injuries [16]. Lower SES is associated with poor housing conditions, lack of safety measures, and delayed access to healthcare, all of which contribute to higher burn severity. These findings highlight the need for socioeconomic support measures, such as financial assistance for safety devices and home improvements, to reduce the risk of severe burns in low-income households.

Lower SES often correlates with a lack of access to educational resources, which can further exacerbate the risk of severe burns. Families with limited financial means may not afford necessary safety devices, such as smoke detectors, stove guards, or fire extinguishers [1]. Additionally, lower SES communities may have less access to healthcare facilities capable of providing specialized burn care, resulting in delayed or suboptimal treatment. Addressing these disparities through targeted public health policies and socioeconomic support programs is essential for reducing the burden of severe burn injuries in low-income populations.

### 4.7. Parental Education Level

Lower parental education levels were associated with more severe burns (mean TBSA of 23%) and prolonged hospital stays. This finding corroborates the research by [22], which emphasizes the role of parental education in burn prevention and immediate response to injuries. Educated parents are more likely to implement effective safety measures and seek timely medical intervention, thereby reducing the severity of burns. Educational programs targeting parents, especially those with lower education levels, can raise awareness about burn prevention and first aid, ultimately improving outcomes for affected children.

Parental education plays a critical role in the prevention and management of pediatric burns. Parents with higher education levels are typically more aware of the potential hazards in the home environment and are better equipped to take preventive measures [22]. They are also more likely to understand the importance of immediate and appropriate medical intervention following a burn injury. Implementing widespread educational programs that target parents with lower education levels can bridge this knowledge gap, promoting safer environments for children and improving overall burn outcomes.

The clinical outcomes of pediatric burn patients in this study highlight several critical aspects that influence recovery and long-term health. The findings underscore the complexity of managing severe burns and the importance of timely, specialized care.

### 4.8. Length of Hospital Stay (LOS)

The average length of hospital stay (LOS) for pediatric burn patients was 12.5 days, with a range from 3 to 45 days. Younger children, particularly those under 3 years, had a notably longer average LOS of 16 days. This extended hospitalization can be attributed to the increased vulnerability of younger children, who require more intensive monitoring and complex medical interventions due to their delicate skin and higher risk of severe burns. Additionally, children from rural areas experienced longer hospital stays (14 days on average) compared to their urban counterparts (11 days). This disparity likely stems from delays in accessing specialized burn care and the need for prolonged treatment to address complications arising from initially inadequate care in less-equipped facilities.

### 4.9. Surgical Interventions

A significant proportion of patients (62%) required surgical interventions, such as debridement and skin grafting. Flame burns necessitated the highest rate of surgical procedures (78%), reflecting the severe and deep tissue damage typically associated with these injuries. Chemical burns also required frequent surgical intervention (67%) due to their potential for deep tissue penetration and complex wound management. Scald burns, while more prevalent, had a moderate need for surgery (55%), and contact burns required the least surgical intervention (48%). These findings indicate the critical role of surgical management in treating severe burns and highlight the need for specialized surgical expertise in pediatric burn care centers.

### 4.10. Complications

Complications were observed in 28% of the cases, with infections (15%), significant scarring (10%), and contractures (3%) being the most common. Infections were particularly prevalent among patients with a TBSA greater than 20%, emphasizing the correlation between the extent of burns and the risk of complications. Patients from lower socioeconomic backgrounds and rural areas exhibited higher complication rates, likely due to delayed treatment and inadequate initial wound care. These findings stress the importance of early and effective intervention, especially for high-risk groups, to prevent complications and improve recovery outcomes.

### 4.11. Mortality

The overall mortality rate in this study was 2.5%, with most fatalities occurring in patients with a TBSA greater than 40%. Younger children, particularly those under 3 years old, had a higher mortality rate of 4%, reflecting their increased susceptibility to severe burns and complications. Flame burns were associated with the highest mortality rate (3.5%), underscoring the need for targeted prevention and intervention strategies for these high-risk injuries. Delays in receiving specialized care, particularly in rural areas, significantly contributed to the higher mortality rates, highlighting the urgent need to improve access to advanced burn care facilities in these regions.

### 4.12. Implications for Practice and Policy

The study’s findings have several important implications for clinical practice and public health policy:

1. Targeted Prevention Strategies: Educational campaigns should be tailored to address the specific risks associated with different age groups, genders, and living environments. For example, programs focusing on kitchen safety and the dangers of hot liquids can significantly reduce scald injuries among young children. Additionally, promoting safe play and risk-averse behaviors in boys could reduce the incidence of severe flame burns.
2. Improving Access to Care: Enhancing healthcare infrastructure in rural areas is crucial. This includes establishing more burn care facilities, improving transportation networks for quicker access to care, and utilizing telemedicine to provide expert consultations in remote regions. Ensuring that rural populations have timely access to advanced burn care can significantly reduce the severity and complications of burn injuries.
3. Educational Programs: Implementing educational programs for parents, particularly those with lower education levels, can raise awareness about burn prevention and first aid. These programs should be accessible and culturally sensitive to effectively reach diverse populations. By educating parents on the importance of home safety measures and proper burn care, the incidence and severity of pediatric burns can be reduced.
4. Socioeconomic Support: Policies aimed at improving the living conditions of lower SES families can mitigate the risk of burn injuries. This includes providing financial assistance for safety devices, home improvements, and ensuring affordable access to healthcare services. Addressing the socioeconomic determinants of health can lead to a significant reduction in burn-related morbidity and mortality.

## 5. Conclusions

Our study provides valuable insights into the severity and clinical outcomes of pediatric burns, highlighting significant predictors of severe burn outcomes, such as rural residence, male gender, flame burns, and lower socioeconomic status.

The findings emphasize the need for targeted prevention strategies, improved access to specialized burn care, and consideration of socioeconomic factors in managing pediatric burns. These findings add to the existing literature by identifying key predictors of severe burn outcomes and providing a foundation for developing targeted interventions.

Future research should focus on refining diagnostic criteria, enhancing data collection methods, and exploring comprehensive care approaches to improve outcomes for pediatric burn patients.

## Figures and Tables

**Table 1:** Demographic and Burn Characteristics Data.

Demographic and Burn Characteristics	Cases
Number of cases	1498
Mean Age (years)	5.8
Gender (Male) (%)	54
Scalds (%)	45
Flame Burns (%)	30
Contact Burns (%)	15
Chemical Burns (%)	5
TBSA < 20% (%)	67.5
TBSA > 20% (%)	32.5
Surgical interventions required (%)	62.5
Mean length of Hospital Stay (days)	11.25
Incidence of Infections (%)	17.5
Incidence of Significant Scarring (%)	11
Incidence of Contractures (%)	3.5
Mortality Rate (%)	2.5

## Data Availability

Datasets used in this study are available upon request.
